# Apple Polyphenols Extract (APE) Alleviated Dextran Sulfate Sodium Induced Acute Ulcerative Colitis and Accompanying Neuroinflammation via Inhibition of Apoptosis and Pyroptosis

**DOI:** 10.3390/foods10112711

**Published:** 2021-11-05

**Authors:** Fang Liu, Xinjing Wang, Yuan Cui, Yan Yin, Dong Qiu, Shilan Li, Xinli Li

**Affiliations:** 1 Department of Nutrition and Food Hygiene, School of Public Health, Medical College of Soochow University, Suzhou 215123, China; 20184247019@stu.suda.edu.cn (F.L.); xjwang@stu.suda.edu.cn (X.W.); 20194247025@stu.suda.edu.cn (Y.C.); 20204247008@stu.suda.edu.cn (Y.Y.); 20205247014@stu.suda.edu.cn (D.Q.); 20205247013@stu.suda.edu.cn (S.L.); 2 Jiangsu Key Laboratory of Preventive and Translational Medicine for Geriatric Diseases, School of Public Health, Soochow University, Suzhou 215123, China

**Keywords:** apple polyphenols extracts, ulcerative colitis, apoptosis, pyroptosis, neuroinflammation

## Abstract

The main aim of this study was to investigate the potent anti-apoptosis and anti-pyroptosis effects of apple polyphenols extract (APE) on dextran sulfate sodium model group (DSS)-induced acute ulcerative colitis (UC) and the protective effect of APE against acute UC-related neuroinflammation and synapse damage. Forty-three C57BL/6 male mice were randomly divided into a control group (CON), a 3% DSS model group (DSS), a 500 mg/(kg·bw·d) APE group (HAP), and a 125 (LD) or 500 (HD) mg/(kg·bw·d) APE treatment concomitantly with DSS treatment group. The results showed that APE significantly ameliorated DSS-induced acute UC through inhibiting intestinal epithelial cell (IEC) apoptosis and the Caspase-1/Caspase-11-dependent pyroptosis pathway, with increased BCL-2 protein expression and decreased protein levels of NLRP3, ASC, Caspase-1/11, and GSDND. Furthermore, APE significantly reduced acute UC-related neuroinflammation and synapse damage, supported by decreased mRNA levels of hypothalamus *Cox-2* and hippocampus *Gfap* and also increased the mRNA levels of hypothalamus *Psd-95*. The increased protein expression of ZO-1 and Occludin improved the intestinal barrier integrity and improved the function of goblet cells by upregulating the protein level of MUC-2 and TTF3 accounted for the beneficial effects of APE on UC-associated neuroinflammation. Therefore, APE might be a safe and effective agent for the management of acute UC.

## 1. Introduction

Ulcerative colitis (UC) is a kind of metabolic disorder whose characteristics are diarrhea, mucus pus, bloody stools, and abdominal pain [1]. In recent years, the incidence of UC has reached a plateau in developed countries but increased in developing countries due to economic development and the uptake of a Westernized diet [2,3,4,5]. Statistical data have shown that the global incidence and prevalence of UC were 1.2–20.3/100,000 and 7.6–245/100,000, respectively [6]. Studies have reported that individuals with UC are susceptible to colon cancer, osteoporosis, venous thromboembolism, cardiovascular disease, and other complications [7,8], thereby causing a heavy burden on economic and health systems worldwide. Currently, the pathogenesis of UC has not yet been fully elucidated and the effects of related drug treatment are a subject of intense discussion.

Despite the precise etiology of UC remaining unknown, studies have shown that excessive intestinal epithelial cell (IEC) apoptosis, which is a cell death modality, plays a key pathogenic role [9,10]. The enhanced apoptosis of IEC disrupts epithelial barrier function [11], which might result in harmful substances in the intestines infiltrating into other tissues, organs, and the blood circulation [12], thereby increasing the risk of UC. Recent studies have reported that the inhibition of IEC apoptosis could improve dextran sulfate sodium (DSS)/tri-nitrobenzene sulphonic acid (TNBS)-induced UC [13,14,15].

In addition to apoptosis, pyroptosis is a newly found programmed cell death process [16], which involves the Caspase-1-dependent canonical pathway and the Caspase-4/5/11-dependent non-canonical pathway [17]. In the canonical pyroptosis pathway, the (NOD)-like receptor family and pyrin domain containing 3 (NLRP3) inflammasome is oligomerized by NLRP3 and the apoptosis-associated speck-like protein (ASC) after receiving certain stimulations such as inflammation and reactive oxygen species, which then activates the Pro-Caspase-1. Subsequently, Cleaved-Caspase-1 triggers the maturation of interleukin-1β (IL-1β) and interleukin-18 (IL-18) and processes gasdermin D (GSDMD) into GSDMD-N, ultimately resulting in pyroptosis [18]. In the Caspase-4/5/11-dependent non-canonical pathway, lipopolysaccharide (LPS) is recognized by human Caspase-4, human Caspase-5, and murine Caspase-11, followed by the direct processing of GSDMD by the activated Caspase-4/5/11, ultimately inducing pyroptosis [16]. Some studies have indicated that pyroptosis plays an important role in UC, as pyroptosis can prompt the release of IL-1β and IL-18 that are essential inflammatory factors for UC initiation [19,20,21]. However, other studies have shown that Caspase-1^-/-^/Caspase-11^-/-^ mice become susceptible to colitis, which suggests a protective effect of pyroptosis against colitis [22,23]. So far, the role of pyroptosis in the development of UC has not been fully elucidated. Therefore, this calls for the further exploration of the relationship between pyroptosis and UC.

Moreover, a recent study found that gut inflammation can potentially affect inflammation markers in the blood and then directly and indirectly trigger neuroinflammatory reactions [24]. Furthermore, excessive neuroinflammation could further cause brain disorders such as depression and anxiety [24,25]. Consequently, gut inflammation could be a novel target for improving UC-related brain disorders.

Apple polyphenols extract (APE), extracted from red Fuji apples, has been reported as having several health-promoting effects, including anti-inflammatory [26], anti-oxidative [26], and anti-tumor effects [27]. In this study, we hypothesized that APE could play a protective role in UC. The hypothesis was supported by Yeganeh et al. [28], who discovered that apple peel polyphenols decreased mitochondrial dysfunction in an experimental UC model. In our previous study [29], we also found that APE could ameliorate DSS-induced UC by suppressing activation of the NF-κB pathway, restoring gut ecology and bile acid metabolism. However, the potential anti-UC role of APE on apoptosis and pyroptosis has not yet been clarified. Therefore, we designed this study to explore, for the first time, the potent anti-apoptosis and anti-pyroptosis effects of APE on acute UC. Moreover, we explored the protective effect of APE against acute UC-related neuroinflammation and synapse damage.

## 2. Materials and Methods

### 2.1. Chemicals and Reagents

APE was obtained from JF-NATURAL (Tianjin, China). Analysis of ingredients of APE showed that the ingredients were phloretin (1.07%), phlorizin (6.55%), procyanidin B2 (4.05%), epicatechin (3.13%), chlorogenic acid (15.2%), catechin (1.36%), quercetin (0.29%), others polyphenols (48.43%), and other ingredients, which accounted for 19.92%.

DSS (MW 36,000–50,000 Da) was purchased from MP Biomedical (Illkirch, France). Enzyme-linked immune sorbent assay (ELISA) kits of C-reactive protein (CRP), tumor necrosis factor-alpha (TNF-α), IL-1β, and IL-18 were purchased from MEIMIAN (Yancheng, China). Primers were synthesized by GENEWIZ (Suzhou, China). All antibodies were obtained from Abcam (Cambridge, UK), except anti-Caspase-1, which was from Immunoway (Plano, American).

### 2.2. Animals

**Figure 1:**
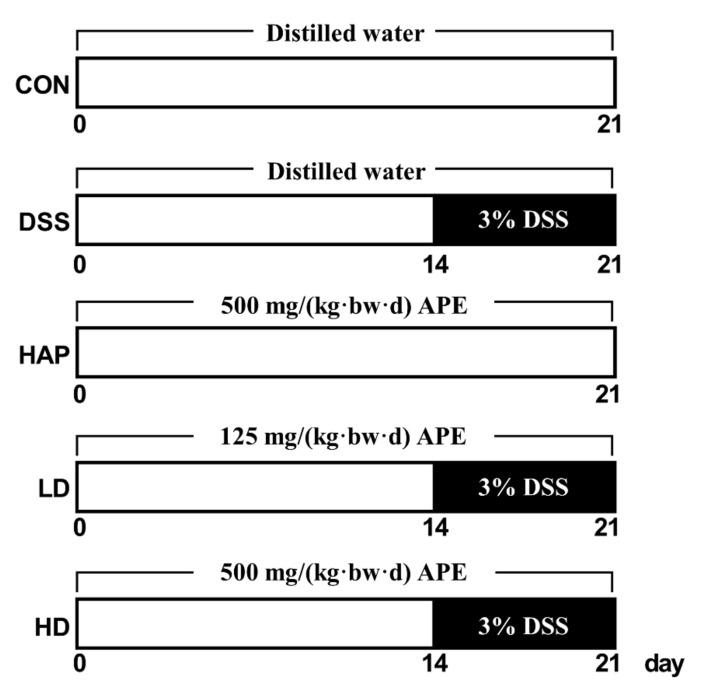
Diagram of study design. APE: apple polyphenols extract. CON: control group. DSS: 3% DSS (dextran sulfate sodium) model group. HAP: 500 mg/(kg.bw.d) APE group. LD: 125 mg/(kg.bw.d) APE + 3% DSS group. HD: 500 mg/(kg.bw.d) APE + 3% DSS group.

Male mice in C57BL/6 background (6–8 weeks old) were obtained from Jihui Laboratory Animal Care Co., Ltd. (Shanghai, China). All mice were randomly assigned into five groups: control group (CON, *n* = 8), DSS group (DSS, *n* = 9), 500 mg/(kg·bw·d) APE group (HAP, *n* = 8), 125 mg/(kg·bw·d) APE + DSS group (LD, *n* = 9), and 500 mg/(kg·bw·d) APE + DSS group (HD, *n* = 9). Mice in HAP, LD, and HD groups were orally treated with 125 or 500 mg/(kg·bw·d) APE, while the mice in CON and DSS groups were gavaged with the same volume of water for 21 days. It is worth noting that three DSS treatment groups (DSS, LD, and HD groups) were administered 3% DSS solution through their drinking water from day 15 to day 21 to induce acute UC, whereas the CON group and HAP group only received the same volume distilled water as DSS. The dose of APE we selected in the present study is from our pilot study, and in our previous study about the alleviated effects of APE on high-fat-diet induced hepatic steatosis, both of doses had also been employed, which demonstrated safe and dose-dependent ameliorated effects. To evaluate the severity of UC, we calculated disease activity index (DAI) scores daily during the modeling period according to the previously reported method [30]. The overall study design is graphically presented in Figure 1. After successful modeling, all mice were sacrificed using pentobarbital under anesthesia. Serum samples were collected for ELISA tests. In addition, colon tissues were harvested and subjected to Western blot analysis, reverse transcriptase polymerase chain reaction (RT-PCR), immunohistochemistry, and other experiments. Spleen tissues were collected and immediately weighed to calculate the spleen index (ratio of spleen weight to body weight). Hypothalamus, hippocampus, and cortex were also harvested for RT-PCR. This study was approved by Soochow University Animal Welfare Committee (Approved No.201904A243), and it conformed to the Guidelines for the Care and Use of Animals.

### 2.3. Histological Analyses of Colon Tissue

We performed hematoxylin and eosin (H & E) staining in accordance with the routine protocols. The histopathological score included the following items: (1) inflammation, (2) mucosal damage, (3) crypt damage, and (4) range of lesions. For inflammation scores: no inflammation (value of 0), mild inflammation (value of 1), moderate inflammation (value of 2), and severe inflammation (value of 3). The mucosal damage scores were determined by the following standards: 0, no mucosal damage; 1, mucous layer damage; 2, submucosa damage; and 3, muscularis and serosa damage. The grade of severity of crypt damage ranked among 0 to 4 where 0 was no crypt damage, 1 was 1/3 crypt damage, 2 was 2/3 crypt damage, 3 was 100% crypt damage, and 4 was 100% crypt damage and epithelium loss. The lesion range scores were assessed using the scoring systems: 0 = 0, 1 = 1–25%, 2 = 26–50%, 3 = 51–75%, and 4 = 76–100%.

### 2.4. Analysis of Serum Samples

We assayed serum contents of CRP, IL-1β, TNF-α, and IL-18 with ELISA kits. Briefly, serum was diluted five times with sample diluent buffer, followed by addition of appropriate enzyme-labeled reagent (100 μL/well) and incubation for 60 min at 37 °C. After washing, 50 μL of chromogen reagent A and 50μL of chromogen reagent B were mixed and added into each well and colored at 37 °C for 15 min. Finally, the stop solution (50 µL) was added, and the absorbance determined at 450 nm.

### 2.5. RT-PCR Analysis

Total RNA from colon, hypothalamus, hippocampus, and cortex was isolated using tissue RNA purification kit, and the synthesis of first-strand cDNA templates was completed using RevertAid first strand cDNA synthesis kit. Next, 0.4 μL of forward primer, 0.4 μL of reverse primer, 9.2 μL of sample, and 10 μL of SYBR were mixed, and used to conduct RT-PCR analysis. The relative expression of gene was calculated using 2^−ΔΔCt^ formula, and GAPDH was selected as the house keeping gene used for normalization. Table 1 shows the primers used.

### 2.6. Immunohistochemistry

We used immunohistochemistry to measure the expression of zonula occludens-1 (ZO-1), Occludin, mucoprotein-2 (MUC2), and trefoil factor 3 (TFF3) proteins in the colon. For antigen retrieval, 4-μm-thick paraffin sections were placed in citrate buffer and microwaved with medium heat for 8 min and heat preservation for 8 min, followed by moderate-low heat for 7 min. After blocking with 3% bovine serum albumin, sections were incubated with anti-ZO-1, anti-Occludin, anti-MUC-2, and anti-TFF3 antibodies. Next, they were mixed with the secondary antibody and incubated. Finally, the sections were stained with diaminobenzidine and counterstained with hematoxylin stain solution. Notably, positive staining presented a brown-yellow color under a microscope (Eclipse E100, Nikon, Tokyo, Japan).

### 2.7. Alcian Blue/Periodic Acid-Schiff (AB-PAS) Staining

For AB-PAS staining, the paraffin-embedded sections were stained with AB-PAS C after dewaxing using xylene and ethanol. Next, the sections were washed with distilled water, followed by acidification with AB-PAS B and staining with AB-PAS A. Finally, the sections were washed with distilled water.

### 2.8. Western Blot Analysis

Protein expression levels of pyroptosis and apoptosis pathway were tested in accordance with a previously described protocol [29].

### 2.9. Terminal Deoxynucleotidyl Transferase-Mediated dUTP Nick End Labeling Assay (TUNEL)

Firstly, 4-μm-thick paraffin sections were incubated with protease K, washed with PBS, and incubated with 3% hydrogen peroxide. They were then rinsed with PBS, followed by immersion in equilibration buffer. Next, the sections were incubated with a mixture of recombinant TdT enzyme, biotin-dUTP labeling mix, and equilibration buffer (1:5:50), and then incubated with streptavidin-HRP. Notably, TUNEL staining and counterstaining procedures were consistent with immunohistochemistry staining. Under a microscope (Eclipse E100, Nikon, Tokyo, Japan), apoptosis-positive cells were brown-yellow in color, while normal cells were blue.

### 2.10. Statistical Analysis

All data were expressed as the mean ± standard error, and all calculations were completed using SPSS version 22. For the parametric data, the ANOVA test followed by LSD for multiple comparisons was utilized to compare differences in the groups. For the non-parametric data such as DAI score and spleen index, the Mann–Whitney rank sum test was used to compare differences in the groups. *p* < 0.05 was considered significant.

## 3. Results

### 3.1. Effect of APE on the Symptoms of DSS-Induced Acute UC

**Figure 2:**
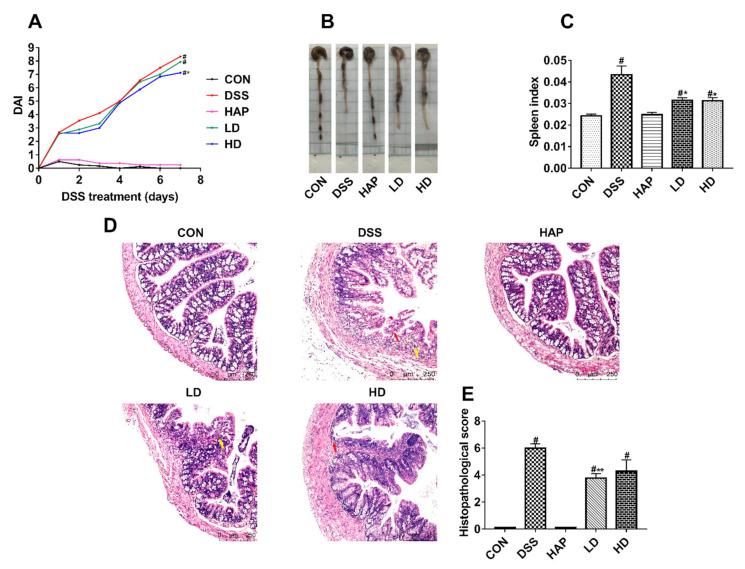
Effect of APE on the symptoms of DSS-induced acute ulcerative colitis (UC): (**A**) Disease activity index (DAI) scores. (**B**) Representative pictures of colon. (**C**) Spleen index. (**D**) Representative image of Hematoxylin and eosin (H&E) staining at 20 × resolution. (**E**) Histopathological scores. Red arrows indicated tissue local mucosal damage. Yellow arrows represented inflammatory cell infiltration. *n* = 8 or 9. # *p* < 0.05 vs. CON group. * *p* < 0.05 vs. DSS group. ** *p* < 0.01 vs. DSS group.

The DAI score was used to assess the severity of UC. Mice treated with 3% DSS presented obviously increased DAI scores, while a high dose of APE intervention showed a protective effect against DSS-caused DAI score increase (Figure 2A). Moreover, the APE intervention groups had a much higher colon length (Figure 2B) and lower spleen index (Figure 2C) compared to the DSS group. HE staining showed severe pathological changes in the colon tissues of the DSS group, including local tissue mucosal damage, inflammatory cell infiltration, etc. However, the administration of a low APE dose significantly ameliorated these changes (Figure 2D,E).

### 3.2. Effect of APE on Pro-Inflammatory Mediators

**Figure 3:**
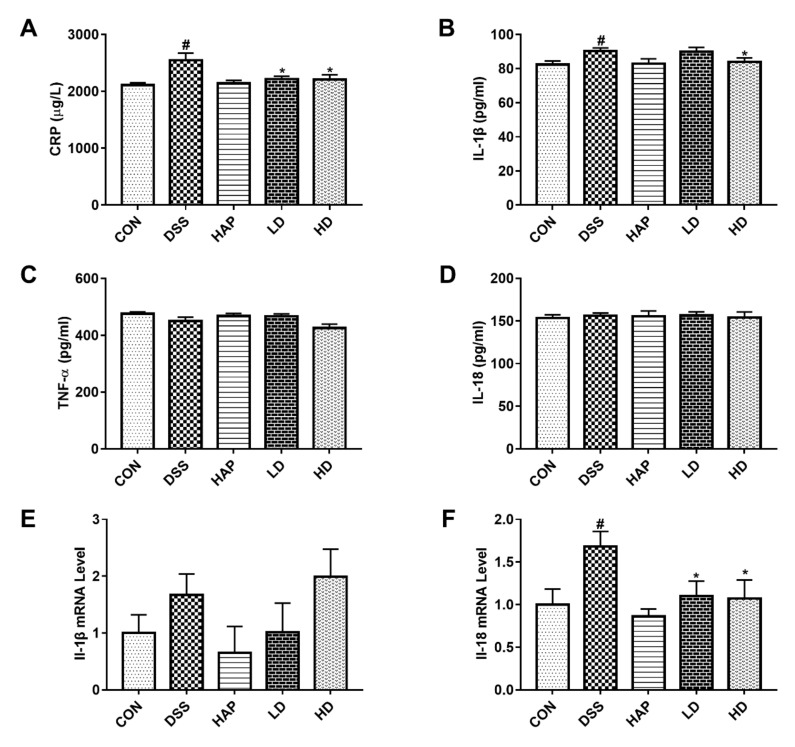
Effect of APE on pro-inflammatory mediators. Serum contents of pro-inflammatory mediators determined using enzyme-linked immune sorbent assay (ELISA) kit: C-reactive protein (CRP) (**A**), interleukin-1β (IL-1β) (**B**), tumor necrosis factor-alpha (TNF-α) (**C**), and interleukin-18 (IL-18) (**D**). The relative mRNA levels of *Il-1β* (**E**) and *Il-18* (**F**) in colon tissue were determined by reverse transcriptase polymerase chain reaction (RT-PCR). *n* = 8 or 9. # *p* < 0.05 vs. CON group. * *p* < 0.05 vs. DSS group.

A previous study positively correlated the severity of UC with the level of some inflammatory mediators [31]. In this study, we used ELISA kits to detect key inflammatory mediators. The results presented that DSS significantly elevated the contents of CRP (Figure 3A) and IL-1β (Figure 3B) in serum. However, low-dose APE ameliorated the DSS-caused production of CRP and high-dose APE alleviated the DSS-mediated increase in CRP and IL-1β. Serum contents of TNF-α (Figure 3C) and IL-18 (Figure 3D) exhibited no significant difference among the five groups.

In addition, we used RT-PCR to assess the mRNA expressions of *Il-1β* and *Il-18* in colon tissue. The results indicated that mice in the DSS group presented higher *Il-1β* and *Il-18* mRNA expressions than those in the CON group (Figure 3E,F), but only the elevation of *Il-18* achieved statistical significance. Moreover, APE intervention significantly inhibited the mRNA expressions of *Il-18* compared to the DSS group, and the difference in *Il-18* mRNA expression between the LD group and HD group did not reach statistical significance.

### 3.3. Effect of APE on Intestinal Tight Junction Proteins

**Figure 4:**
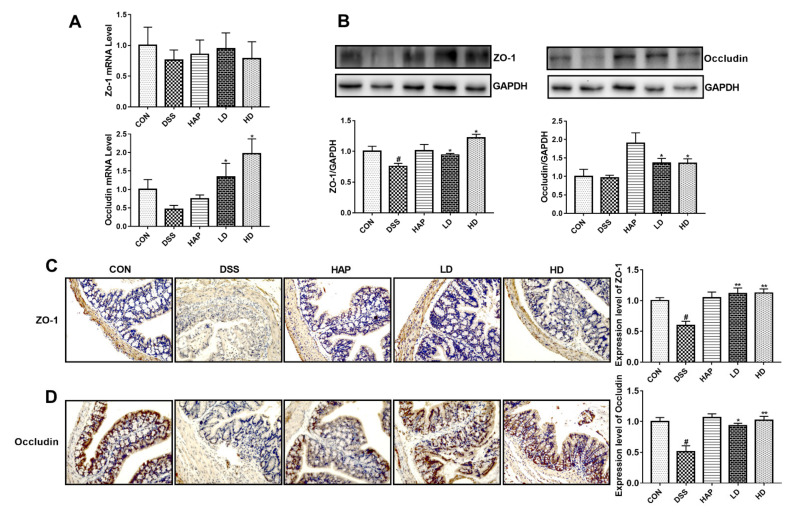
Effect of APE on intestinal tight junction proteins: (**A**) The mRNA levels of zonula occludens-1 (*Zo-1*) and *Occludin* in colon tissue were determined by RT-PCR. (**B**) The protein levels of ZO-1 and Occludin in colon tissue were measured by Western blot. The protein levels of ZO-1 (**C**) and Occludin (**D**) were determined by immunohistochemistry. *n* = 8 or 9. # *p* < 0.05 vs. CON group. * *p* < 0.05 vs. DSS group. ** *p* < 0.01 vs. DSS group.

The mRNA expressions of Zo-1 and Occludin and the protein expression of Occludin in the DSS group mice were not altered compared to the CON group, while ZO-1 protein expression was obviously lower in the DSS group (Figure 4A,B). APE administration significantly increased the Occludin mRNA expression and the ZO-1 and Occludin protein expressions compared to those in the DSS group, with no significant difference between the LD group and the HD group.

Furthermore, we examined the expressions of intestinal tight junction proteins using immunohistochemistry. As expected, DSS treatment resulted in a marked decrease in the protein expressions of ZO-1 (Figure 4C) and Occludin (Figure 4D) than those in the CON group, and the APE administration significantly reversed the effects on ZO-1 and Occludin. Notably, differences in the protein expressions of ZO-1 and Occludin between the LD and HD groups did not reach statistical significance.

### 3.4. Effect of APE on the Function of Goblet Cells

**Figure 5:**
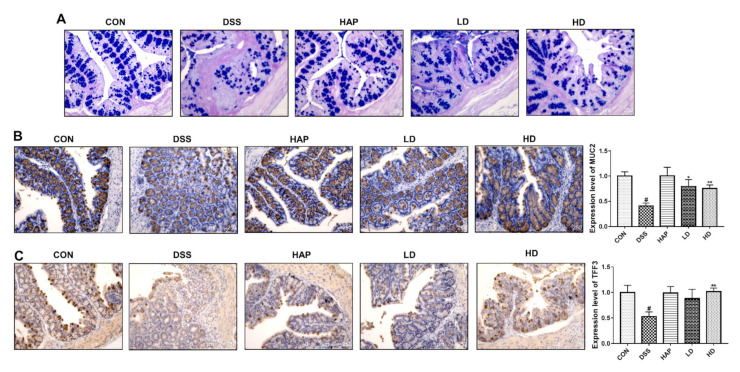
Effect of APE on the function of goblet cells: (**A**) Representative image of alcian blue/periodic acid-schiff (AB-PAS) staining at 20 × resolution. (**B**) The protein level of mucoprotein-2 (MUC2) was determined by immunohistochemistry. (**C**) The proteins level of trefoil factor 3 (TFF3) was determined by immunohistochemistry. *n* = 8 or 9. # *p* < 0.05 vs. CON group. * *p* < 0.05 vs. DSS group. ** *p* < 0.01 vs. DSS group.

Next, we explored whether the APE administration improved the function of goblet cells in DSS-induced acute colitis. AB-PAS staining showed that the quantity of goblet cells was significantly reduced by DSS (Figure 5A). However, the elevated quantity of goblet cells was observed after APE intervention compared with the DSS group. We also determined the effects of APE administration on the production of intestinal MUC-2 and TFF3. Immunohistochemical results indicated that, compared to the CON group, the colon proteins level of MUC-2 and TFF3 were markedly reduced in the DSS group (Figure 5B,C). In addition, the protein level of MUC-2 was markedly increased in the LD and HD groups compared to the DSS group. Meanwhile, a high APE dose also significantly increased the protein level of TFF3 compared to the DSS group. Differences in the protein levels of MUC-2 and TFF3 between the LD group and the HD group were not statistically significant.

### 3.5. Effect of APE on IEC Apoptosis

**Figure 6:**
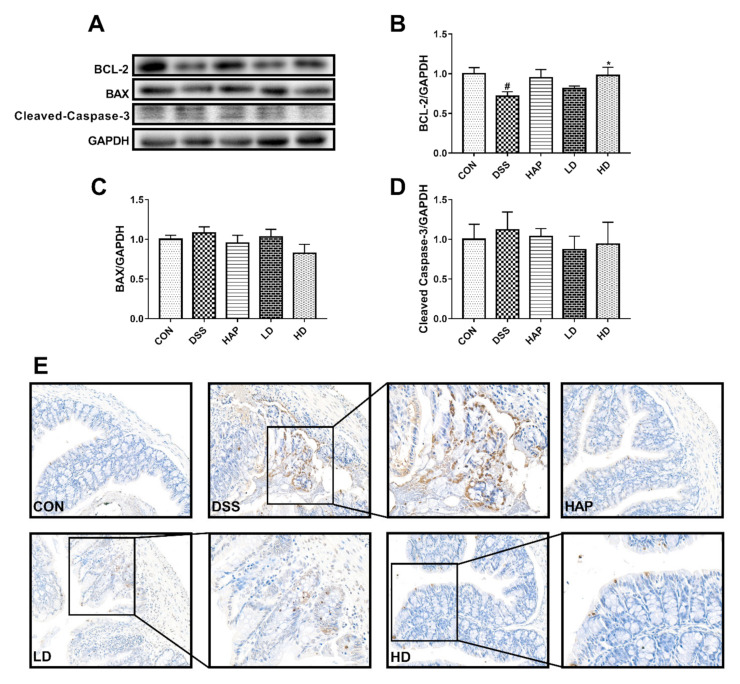
Effect of APE on intestinal epithelial cell (IEC) apoptosis: The levels of B-cell lymphoma 2 (BCL-2) (**B**), BCL2-associated X (BAX) (**C**) and Cleaved-Caspase-3 (**D**) in colon tissue were measured by Western blot (**A**). (**E**) Representative pictures of terminal deoxynucleotidyl transferase-mediated dUTP nick end labeling assay (TUNEL) staining. *n* = 8 or 9. # *p* < 0.05 vs. CON group. * *p* < 0.05 vs. DSS group.

The results of the Western blot showed that DSS dramatically decreased B-cell lymphoma 2 (BCL-2) protein expression (Figure 6A,B), whereas this abnormal decrease was improved by intervention with high-dose APE. The differences in the protein expression of BCL2-associated X (BAX) (Figure 6C) and Cleaved-Caspase-3 (Figure 6D) among the five groups were not statistically significant.

To further verify our results, the TUNEL method was used to detect IEC apoptosis. Figure 6E showed that apoptotic IEC was rarely observed in the colon obtained from mice in the CON group. DSS upregulated the number of TUNEL-positive cells in the colon compared to the CON group, and APE administration ameliorated the increased number of TUNEL-positive cells.

### 3.6. Effect of APE on the Pyroptosis Signaling Pathway

**Figure 7:**
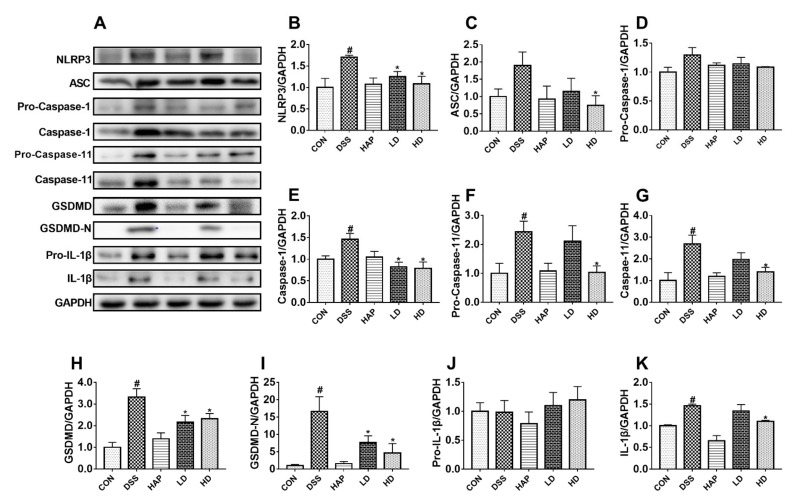
Effect of APE on the pyroptosis signaling pathway: The levels of (NOD)-like receptor family and pyrin domain containing 3 (NLRP3) (**B**), apoptosis-associated speck-like protein (ASC) (**C**), Pro-Caspase-1 (**D**), Caspase-1 (**E**), Pro-Caspase-11 (**F**), Caspase-11 (**G**), gasdermin D (GSDMD) (**H**), GSDMD-N **(I)**, Pro-IL-1β (**J**) and IL-1β (**K**) in colon tissue were measured by Western blot **(A)**. *n* = 8 or 9. # *p* < 0.05 vs. CON group. * *p* < 0.05 vs. DSS group.

To study the suppressive effects of APE on pro-inflammatory mediators, the key proteins in the pyroptosis signaling pathway were evaluated. As shown in Figure 7A–K, the protein expression levels of NLRP3, Caspase-1, Pro-Caspase-11, Caspase-11, GSDMD, GSDMD-N, and IL-1β measured by Western blot were significantly upregulated in the DSS group than the levels in the CON group. However, a low APE dose significantly downregulated the protein expression level of NLRP3, Caspase-1, GSDMD, and GSDMD-N compared to the DSS group. On the other hand, a high APE dose significantly decreased the protein expression level of NLRP3, ASC, Caspase-1, Pro-Caspase-11, Caspase-11, GSDMD, GSDMD-N, and IL-1β compared to the DSS group. Furthermore, a comparison between the LD group and the HD group indicated that the differences in the protein expressions of the pyroptosis signaling pathway intermediates were not statistically significant.

### 3.7. Effect of APE on Acute UC-Related Neuroinflammation and Synapse Damage

**Figure 8:**
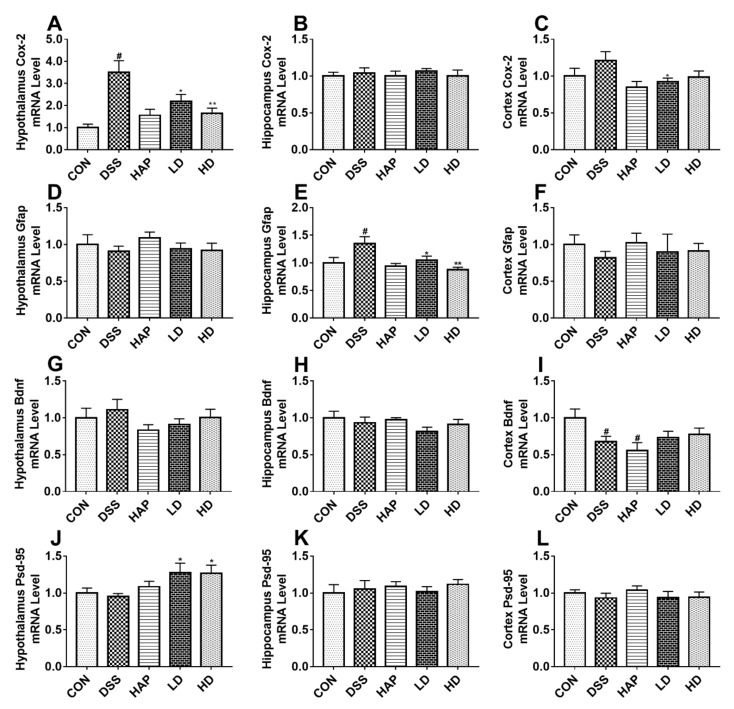
Effect of APE on acute UC-related neuroinflammation and synapse damage: The mRNA levels of cyclooxygenase-2 (*Cox-2*) in hypothalamus (**A**), hippocampus (**B**), and cortex (**C**). The mRNA levels of glial fibrillary acidic protein (*Gfap*) in hypothalamus (**D**), hippocampus (**E**), and cortex (**F**). The mRNA levels of brain derived neurotrophic factor (*Bdnf*) in hypothalamus (**G**), hippocampus (**H**), and cortex (**I**). The mRNA levels of postsynaptic-density protein 95 (*Psd-95*) in hypothalamus (**J**), hippocampus (**K**), and cortex (**L**). *n* = 8 or 9. # *p* < 0.05 vs. CON group. * *p* < 0.05 vs. DSS group. ** *p* < 0.01 vs. DSS group.

A previous study reported that colitis can alter brain function [24]. Based on this, we further determined the mRNA levels of cyclooxygenase-2 (*Cox-2*), glial fibrillary acidic protein (*Gfap*), brain-derived neurotrophic factor (*Bdnf*)*,* and postsynaptic-density protein 95 (*Psd-95*) in the hypothalamus, hippocampus, and cortex. Although DSS obviously increased the mRNA expression of *Cox-2* in the hypothalamus as compared to the CON group (Figure 8A), APE was able to reverse this change. There were no significant differences in the *Cox-2* mRNA levels in the hippocampus among the five groups (Figure 8B). Furthermore, low-dose APE strongly decreased the mRNA expression level of *Cox-2* in the cortex than that in the DSS group (Figure 8C).

There were no significant changes in *Gfap* mRNA expression levels in the hypothalamus among the five groups (Figure 8D). The mRNA expression of hippocampus *Gfap* resembled the mRNA expression of hypothalamus *Cox-2* (Figure 8E). In the cortex, the *Gfap* mRNA expression trend was similar to that in the hypothalamus (Figure 8F).

As shown in Figure 8G,H, *Bdnf* expression levels in the hypothalamus and hippocampus did not differ among all groups. DSS significantly lowered the mRNA expression of *Bdnf* in the cortex. Moreover, intervention with APE did not remarkably elevate the mRNA expression of *Bdnf* in the colitis cortex tissue (Figure 8I).

Moreover, the mRNA expression of *Psd-95* in the hypothalamus presented a downward trend in the DSS group compared to the CON group (Figure 8J), but it was remarkably higher in the LD and HD groups. However, there was no significant difference in the mRNA expressions of *Psd-95* between the LD group and the HD group. Furthermore, there were no significant differences in the *Psd-95* expression in the hippocampus (Figure 8K) and cortex (Figure 8L) among all groups.

## 4. Discussion

The main findings of this study can be summarized as follows: APE could (a) moderately protect intestinal tight junction proteins; (b) significantly improve the function of goblet cells and increase the protein expression of MUC-2 and TFF3; (c) effectively suppress DSS-induced apoptosis; (d) significantly improve DSS-caused pyroptosis; and (e) significantly ameliorate acute UC-related neuroinflammation and synapse damage. To our knowledge, this is the first study that has explored the novel molecular mechanisms of APE against acute UC by inhibiting apoptosis and pyroptosis. It is also the first demonstration that acute UC-related neuroinflammation and synapse damage can be ameliorated by APE, thereby providing new insights into the pathophysiological role of UC prevention by APE.

Tight junction proteins restrict the permeability and protect the stability of the intestinal barrier [32]. ZO-1 and Occludin are very important components for intestinal barrier integrity, while they are decreased after UC [33]. Our results illustrated that APE administration could increase the expression of tight junction proteins, indicating that APE may be an effective natural agent for the prevention of acute UC. Furthermore, we also determined the protein expression of MUC-2 and TFF3, which are mainly secreted by goblet cells. A previous study reported that the increased expression of MUC-2 and TFF3 protects the integrity of the mucosal barrier [34]. Our results also reflected that the role of APE in protecting intestinal barrier integrity might be associated with its regulation of MUC-2 and TFF3 secretion.

Apoptosis in the intestinal epithelium has been considered as one of the hallmarks of UC [15]. Previous studies have also reported that inappropriate IEC apoptosis is highly associated with increased intestinal permeability [35,36]. Under normal conditions, BCL-2 and BAX are in a relatively stable state and regulate each other, which means that related apoptosis may be induced once this stable state is broken [37]. Caspase-3, known as the “death protease”, is not only a key protease for mammalian cell apoptosis but is also a key downstream effector of multiple apoptotic pathways [38]. It has been reported that apoptosis-related proteins such as Caspase-3 and BAX are significantly increased, and BCL-2 expression is markedly decreased in animal UC models [39]. Therefore, it is vital to improve apoptosis for UC treatment. Herein, we used Western blot analysis and TUNEL staining to detect IEC apoptosis in colon tissues. The Western blot results demonstrated that the protein expression of BCL-2 in colon tissue was markedly downregulated by DSS. However, APE intervention upregulated BCL-2 protein level, indicating that the protective effects of APE in the amelioration of acute UC relied on its inhibition of apoptosis. Moreover, TUNEL assay results confirmed that APE could inhibit the apoptosis of IEC.

The NLRP3 inflammasome, consisting of NLRP3, ASC, and Caspase-1, relates to perceiving microbes and endogenous hazard signals [40]. Several studies have revealed that NLRP3 inflammasome, a key sensor in the pyroptosis signaling pathway, could be activated in the colitis model [41,42,43]. In this study, the significantly increased protein levels of NLRP3, Caspase-1, GSDMD, GSDMD-N, and IL-1β, combined with the higher expression of serum *IL-1β* and colon *Il-18* in DSS-treated mice, suggested the activation of NLRP3 inflammasome and the subsequent Caspase-1-dependent pyroptosis signaling pathway. On the other hand, the inhibited upward trend of NLRP3, ASC, Caspase-1, GSDMD, GSDMD-N, and IL-1β by APE administration, along with the lower expression of serum *IL-1β* and colon *Il-18*, demonstrated that APE could effectively ameliorate DSS-induced acute UC via inhibiting NLRP3 inflammasome-dependent pyroptosis.

In addition to the classical Caspase-1-dependent pyroptosis pathway, the non-classical Caspase-11-dependent pyroptosis pathway plays an important value in processes such as DSS-induced UC [44,45]. Active Caspase-11 is able to directly promote pyroptosis by cleaving GSDMD, and it can also trigger the activation of the NLRP3 inflammasome and maturation of IL-1β and IL-18 [46]. Shen-Ling-Bai-Zhu-San, a traditional Chinese medicine, has been reported to improve DSS-induced UC by inhibiting Caspase-11-mediated pyroptosis [44]. Our results were consistent with the above published study because the significantly increased protein expressions of Pro-Caspase-11 and Caspase-11 in the DSS group were reversed after APE intervention. This suggested that APE could also improve DSS-induced acute UC through inhibiting Caspase-11-dependent pyroptosis.

In addition, psychological symptoms, particularly depression and anxiety, are prevalent in patients with UC, which may further affect patient quality of life [47]. Addolorato et al. [48], found that the percentage of subjects with depression or state anxiety was significantly higher in UC groups (50.0% and 63.9%, respectively) than in control subjects (11.1% and 22.2%, respectively). The specific causes of UC-associated psychological symptoms have not been fully clarified, but they might be partly explained by gut inflammation that triggers neuroinflammation, thereby causing brain disorders [48,49]. As reported, the gut inflammation caused by DSS might impact brain function through the gut–brain axis. Our study found that the mRNA expression of *Cox-2* was significantly increased in the hypothalamus, while *Gfap* mRNA expression was upregulated in the hippocampus. This combined with the increased mRNA expression of *Il-18* and elevated protein expression of IL-1β in colon tissues after DSS intervention, supported the fact that DSS-induced acute UC could cause neuroinflammation in a bottom-up manner. APE intervention significantly reversed the above changes, suggesting that APE has the potential to ameliorate acute UC-related neuroinflammation. Furthermore, the APE intervention increased the mRNA level of *Psd-95*, a well-recognized marker of synapse damage, which further indicated the protective effect of APE on psychological symptoms such as depression. However, future studies should elucidate the specific mechanism.

This study had some limitations. First, we did not determine the serum LPS concentrations due to the limited quantity of serum samples. However, there is one study reported that phloretin, the typical ingredient of APE, significantly reduced serum LPS content relative to the DSS model group [50], which indirectly supported the role of APE in decreasing the LPS content, then further inhibiting LPS-activated Caspase-11-dependent pyroptosis to a certain extent. It is no doubt that the determination of serum LPS concentrations after APE treatment is more convincing. Second, we did not collect data on psychological symptoms. Third, the absence of a positive control drug group was also a big weakness of this study. Fourth, as a mixture, in addition to polyphenols, there were some other ingredients such as moisture, protein, and glucose in APE. Although these other ingredients might not confer protective effects against DSS-induced acute UC because of low content, they might interfere with the effects of apple polyphenols. Fifth, the mRNA level of *Il-18* was significantly altered in the colon, but the protein level showed no significant variation in the serum. This might be due to mRNA changes occurring faster than protein changes. In addition, we did not set the medium-dose group so that we could not well explain the dose–response relationship in this experiment.

## 5. Conclusions

**Figure 9:**
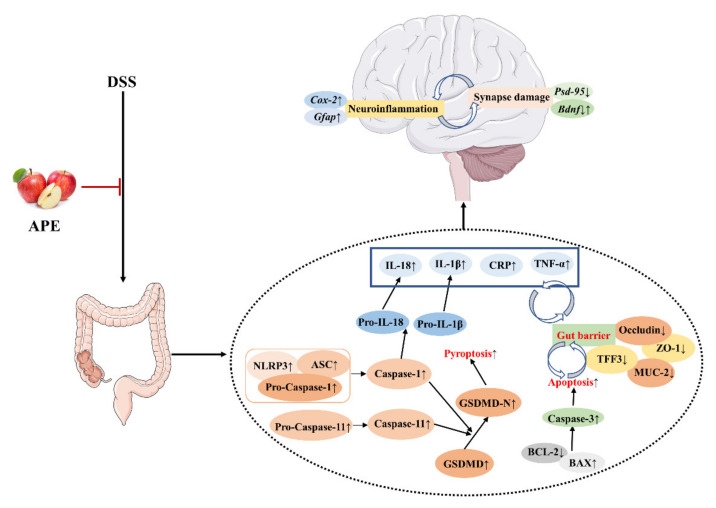
Potential mechanisms that APE protected in DSS-induced acute UC in C57BL/6 male mice.

In summary, our results suggested that APE might be an effective preventive agent against DSS-induced acute UC. The exploration of the underlying mechanism indicated that APE administration ameliorated acute UC by protecting intestinal barrier integrity and inhibiting apoptosis and pyroptosis (Figure 9). Moreover, we found that APE could improve acute UC-related neuroinflammation and synapse damage (Figure 9). Therefore, APE may be a safe and effective agent for UC management.

## Figures and Tables

**Table 1:** Primer sequences of the genes used for the qRT-PCR.

Gene	Forward Primer	Reverse Primer
*Il-1β*	GCGTTGGCCCTCGGTCGAGTTCTA	CGGTCATATAGGAGCCCTTG
*Il-18*	GACTCTTGCGTCAACTTCAAGG	CAGGCTGTCTTTTGTCAACGA
*Zo-1*	ACCACCAACCCGAGAAGAC	CAGGAGTCATGGACGCACA
*Occludin*	TTGAAAGTCCACCTCCTTACAGA	CCGGATAAAAAGAGTACGCTGG
*Gfap*	TCTATGAGGAGGAAGTTCGAGA	TGCAAACTTAGACCGATACCA
*Cox-2*	CAGACAACATAAACTGCGCCTT	GATACACCTCTCCACCAATGACC
*Bdnf*	CTCCGCCATGCAATTTCCACT	GCCTTCATGCAACCGAAGTA
*Psd-95*	TCTGTGCGAGAGGTAGCAGA	AAGCACTCCGTGAACTCCTG
*Gapdh*	AGGTCGGTGTGAACGGATTTG	GGGGTCGTTGATGGCAACA

Abbreviations: *Il-1β*, interleukin-1β; *Il-18*, interleukin-18; *Zo-1*, zonula occludens-1; *Gfap*, glial fibrillary acidic protein; *Cox-2*, cyclooxygenase-2; *Bdnf*, brain derived neurotrophic factor; *Psd-95*, postsynaptic-density protein 95.

## Data Availability

The data presented in this study are available on request from the corresponding author.
